# Protein language model embeddings improve HIV drug resistance prediction: a comprehensive benchmark with attention-based interpretability

**DOI:** 10.1093/bioinformatics/btag260

**Published:** 2026-05-09

**Authors:** Hayden Farquhar

**Affiliations:** Independent Researcher, Finley, New South Wales, Australia

## Abstract

**Motivation:**

Accurate prediction of HIV drug resistance from viral sequences is critical for optimizing antiretroviral therapy. Traditional machine-learning approaches using binary mutation encoding achieve strong accuracy but may fail to capture epistatic interactions and structural features relevant to resistance mechanisms. Protein language models (PLMs) offer learned representations encoding evolutionary and structural information, but have not been systematically benchmarked for HIV resistance prediction across the modern antiretroviral drug set.

**Results:**

We evaluated ESM-2 (650 M parameters) with attention-weighted pooling for predicting resistance to 18 drugs across three classes (protease inhibitors, NRTIs, NNRTIs) on the Stanford HIVDB dataset (*n* = 6308 sequences). Attention-weighted ESM-2 embeddings significantly outperformed XGBoost baselines with binary mutation encoding (mean AUC 0.968 versus 0.955, *P* = .0017), with gains across 15 of 18 drugs and the largest improvements for drugs with complex resistance patterns. Attention weights showed 2.48-fold enrichment at known drug-resistance-mutation positions (*P* < .05 for 63% of drugs; NRTIs strongest at 4.20-fold). External validation on a 20% holdout showed minimal degradation (AUC 0.934). Benchmarking against ESM C 600M and ESM-1v confirmed performance is robust to PLM choice (mean AUC 0.942–0.946 across backbones). Performance was maintained across HIV-1 subtypes (B 0.924; B-divergent 0.900; non-B 0.884) and a temporal holdout (AUC 0.930).

**Availability and implementation:**

Source code is available at https://github.com/hayden-farquhar/HIV-ESM-2 under an MIT license and archived at https://doi.org/10.5281/zenodo.19466629. Stanford HIVDB genotype–phenotype data are publicly available at https://hivdb.stanford.edu/.

## 1 Introduction

### 1.1 The global HIV burden and antiretroviral therapy

Human immunodeficiency virus (HIV) infection remains one of the most significant global public health challenges of our time. According to the most recent UNAIDS estimates, approximately 40.8 million people were living with HIV worldwide in 2024, with 1.3 million new infections and 630 000 AIDS-related deaths occurring that year alone (UNAIDS 2025) (https://www.unaids.org/en/resources/fact-sheet). Since the beginning of the epidemic, over 91 million people have acquired HIV infection, and more than 44 million have died from AIDS-related illnesses.

The introduction of combination antiretroviral therapy (ART) in the mid-1990s fundamentally transformed HIV from a uniformly fatal infection into a manageable chronic condition. Modern ART regimens, typically consisting of three or more drugs targeting different viral proteins, can suppress viral replication to undetectable levels, preventing disease progression and virtually eliminating onward transmission (Bekker et al. 2023). As of 2024, approximately 31.6 million people were accessing ART globally, representing 77% of all people living with HIV (WHO 2025) (https://www.who.int/teams/global-hiv-hepatitis-and-stis-programmes/hiv/strategic-information/hiv-data-and-statistics). Life expectancy for people with HIV on effective treatment now approaches that of the general population in many settings.

However, the emergence and accumulation of drug resistance mutations (DRMs) pose a persistent threat to treatment success. Drug resistance can arise through two primary mechanisms: acquired resistance, which develops in individuals receiving antiretroviral treatment due to incomplete viral suppression; and transmitted resistance, where drug-resistant viral variants are passed to newly infected individuals (Cortez and Maldarelli 2011). The high mutation rate of HIV reverse transcriptase (RT), combined with the large viral population in infected individuals, creates substantial genetic diversity that allows rapid adaptation to selective drug pressure (Rambaut et al. 2004).

### 1.2 Molecular basis of HIV drug resistance

HIV-1 protease and RT are the primary targets of current antiretroviral drugs and the main sites where resistance mutations accumulate. HIV-1 protease is a 99-amino acid aspartic protease that functions as a homodimer, with its active site containing the characteristic Asp-Thr-Gly catalytic triad (Weber and Agniswamy 2009). This enzyme is essential for cleaving the Gag and Gag-Pol polyproteins to produce mature viral proteins required for infectious virion assembly. Protease inhibitors (PIs) bind to the active site cavity, mimicking the transition state of substrate cleavage and blocking enzymatic activity.

Resistance to PIs develops through a complex interplay of primary and secondary mutations. Primary mutations typically occur at residues directly contacting the inhibitor within the active site, reducing binding affinity. Secondary or accessory mutations occur at more distal sites and may act through multiple mechanisms: compensating for reduced catalytic efficiency caused by primary mutations, altering protein dynamics to indirectly affect inhibitor binding, or modifying dimer stability (Schiffer and Weber 2011, Paulsen et al. 2022). The distributed nature of protease resistance, involving mutations throughout the enzyme structure, presents challenges for resistance prediction methods that rely solely on local sequence features.

HIV-1 RT is a heterodimeric enzyme consisting of p66 and p51 subunits that catalyses conversion of the viral RNA genome into double-stranded DNA. RT is targeted by two mechanistically distinct drug classes: nucleoside/nucleotide RT inhibitors (NRTIs) that act as chain terminators upon incorporation into the growing DNA strand, and non-nucleoside RT inhibitors (NNRTIs) that bind to an allosteric pocket and induce conformational changes that impair polymerase function (Sarafianos et al. 2009, Das et al. 2012).

NRTI resistance emerges through two primary mechanisms: enhanced discrimination, where mutations increase the enzyme’s ability to distinguish between NRTIs and natural nucleotides; and excision, where mutations enhance the removal of already-incorporated NRTIs through ATP-mediated pyrophosphorolysis (Das et al. 2012). The canonical M184V mutation, which confers high-level resistance to lamivudine (3TC) and emtricitabine (FTC), exemplifies the discrimination mechanism. Thymidine analog mutations (TAMs) at positions 41, 67, 70, 210, 215, and 219 enhance excision activity and confer cross-resistance to multiple NRTIs. NNRTI resistance typically involves mutations in or near the allosteric binding pocket that reduce drug binding affinity while preserving polymerase function.

### 1.3 Computational approaches to drug resistance prediction

The complexity of HIV drug resistance, with its combinatorial mutation patterns and drug-specific effects, has motivated extensive development of computational prediction methods. The Stanford HIV Drug Resistance Database (HIVdb) represents the gold standard for genotypic resistance interpretation, providing a rules-based algorithm that assigns penalty scores to individual mutations and mutation combinations (Liu and Shafer 2006, Shafer 2006). While clinically validated and widely used, rules-based systems require continuous expert curation and may not optimally capture complex epistatic interactions between mutations.

Machine learning approaches have been extensively explored as alternatives or supplements to rules-based interpretation. Early studies employed support vector machines (SVMs), random forests, and artificial neural networks trained on genotype–phenotype datasets from the Stanford HIVdb to predict quantitative resistance levels or binary resistance classifications (Beerenwinkel et al. 2003, Rhee et al. 2006). These methods typically encode sequences as binary mutation vectors relative to a reference strain, providing interpretable features directly corresponding to specific amino acid substitutions.

More recent work has explored deep learning architectures for HIV resistance prediction. Steiner et al. (2020) systematically evaluated convolutional neural networks (CNNs), recurrent neural networks (RNNs), and hybrid architectures, demonstrating competitive performance with traditional methods while enabling end-to-end learning from raw sequences. Blassel et al. (2021a) applied gradient boosting and random forests to large-scale HIV sequence data, identifying six novel mutations significantly associated with resistance. Pawar et al. (2018) and Shah et al. (2020) integrated structural information with sequence features to improve prediction of PI resistance.

Despite these advances, traditional approaches share a common limitation: they rely on explicit feature engineering that may not fully capture the complex sequence–structure–function relationships underlying drug resistance. Binary mutation encoding treats each position independently, potentially missing important dependencies between sites. Structural features require solving or modeling the protein structure, adding computational complexity and potentially introducing errors for novel variants.

### 1.4 Protein language models: a new paradigm

Protein language models (PLMs) have transformed computational biology by providing learned representations that encode evolutionary, structural, and functional information without explicit supervision. These models, inspired by advances in natural language processing, are trained on massive protein sequence databases using self-supervised objectives such as masked language modeling (Elnaggar et al. 2022, Lin et al. 2023). By learning to predict masked amino acids from their sequence context, PLMs implicitly capture patterns of evolutionary conservation, co-evolution, and structural constraints.

The evolutionary scale modeling (ESM) family of models, developed by Meta AI, has demonstrated remarkable capabilities across diverse protein prediction tasks. ESM-2, the current state-of-the-art, is available in variants ranging from 8 million to 15 billion parameters (Lin et al. 2023). The 650-million parameter variant (esm2_t33_650M_UR50D) represents an effective balance between representation quality and computational efficiency. ESM-2 generates 1280-dimensional embedding vectors for each residue position, capturing contextual information about each amino acid’s structural and functional role.

PLMs have shown particular promise for variant effect prediction, where embeddings capture information relevant to distinguishing deleterious from benign mutations. Studies have demonstrated that ESM embeddings improve prediction of protein stability changes, enzyme activity effects, and fitness landscape characterization (Meier et al. 2021). The attention mechanisms within transformer-based PLMs provide an additional interpretability advantage: attention weights can reveal which sequence positions the model considers most important for a given prediction, potentially highlighting functionally relevant residues.

Applications of PLMs to antimicrobial resistance prediction have begun to emerge. Wu et al. (2023) developed PLM-ARG for antibiotic resistance gene identification using ESM-2 embeddings. García-Jacas et al. (2024) combined ESMFold-predicted structures with ESM-2 embeddings for antimicrobial peptide prediction. Kutluay et al. (2023) demonstrated that transfer learning from pre-trained PLMs improves antimicrobial peptide classification. These successes in related domains suggest that PLMs may similarly benefit HIV drug resistance prediction.

### 1.5 Knowledge gap and study objectives

PLMs have demonstrated capabilities across diverse prediction tasks, yet their systematic application to HIV drug resistance prediction remains unexplored. This represents a significant methodological gap for several reasons. First, HIV resistance involves subtle structural changes that may be captured by PLM embeddings but missed by binary mutation encoding. Second, the attention mechanisms in PLMs could provide interpretable insights into which sequence positions drive resistance predictions. Third, PLMs may encode evolutionary information relevant to distinguishing resistance-conferring mutations from benign polymorphisms.

In this study, we address this gap through a comprehensive evaluation of ESM-2 embeddings for HIV drug resistance prediction. Our specific objectives were to: (1) benchmark ESM-2 representations against traditional binary mutation encoding across all three major antiretroviral drug classes; (2) develop an attention-weighted pooling mechanism that learns position-specific importance for aggregating per-residue embeddings; (3) assess biological interpretability by analysing enrichment of attention weights at known DRM positions; (4) ensure clinical reliability through external validation and calibration analysis; and (5) identify novel high-attention positions that may represent uncharacterized resistance-associated sites; (6) assess the robustness of findings by comparing ESM-2 against alternative PLM backbones (ESM C 600M and ESM-1v); and (7) evaluate generalizability across HIV-1 subtypes and through temporal holdout validation.

## 2 Materials and methods

### 2.1 Data sources

HIV-1 protease and RT sequences with matched phenotypic resistance data were obtained from the Stanford HIV Drug Resistance Database (HIVDB; https://hivdb.stanford.edu/). The database contains sequences from clinical isolates along with in vitro susceptibility measurements expressed as fold-change (FC) in IC_50_ relative to wild-type reference for each drug, determined using the PhenoSense assay (Petropoulos et al. 2000). We used established clinical cutoffs to dichotomize resistance: sequences with FC above the resistance threshold were labeled resistant, and those below were labeled susceptible.

The final dataset comprised 6308 unique sequences across three drug classes: 2171 PI sequences tested against eight drugs [atazanavir (ATV), darunavir (DRV), fosamprenavir (FPV), indinavir (IDV), lopinavir (LPV), nelfinavir (NFV), saquinavir (SQV), tipranavir (TPV)]; 1867 nucleoside RT inhibitor (NRTI) sequences tested against six drugs [lamivudine (3TC), abacavir (ABC), zidovudine (AZT), stavudine (D4T), didanosine (DDI), tenofovir (TDF)]; and 2270 non-nucleoside reverse transcriptase inhibitor (NNRTI) sequences tested against four drugs [efavirenz (EFV), etravirine (ETR), nevirapine (NVP), rilpivirine (RPV)].

Known DRMs were extracted from the International Antiviral Society-USA (IAS-USA) 2022 guidelines (Wensing et al. 2022) for interpretability validation. This curated list represents the current consensus on clinically significant resistance mutations based on in vitro susceptibility data, clinical correlation studies, and expert review.

### 2.2 Sequence preprocessing

Sequences were aligned to the HXB2 reference strain (GenBank accession K03455). Stop codons and ambiguous amino acids were replaced with the most common residue at each position based on the training set distribution. Sequences with more than 10% missing positions were excluded. For ESM-2 processing, sequences were formatted according to model requirements using the standard amino acid alphabet (20 canonical residues). Internal stop codons, which occur at low frequency in clinical sequences due to sequencing errors or biological artefacts, were replaced with the consensus amino acid to avoid tokenization errors.

### 2.3 Baseline models

Traditional machine learning baselines employed binary mutation encoding, where each position was represented as a binary vector indicating the presence or absence of mutations relative to the wild-type consensus. This encoding captures which positions have changed but not the specific amino acid substitution, following conventions from prior HIV resistance studies. XGBoost classifiers (Chen and Guestrin 2016) were trained with default hyperparameters and five-fold stratified cross-validation. Performance was evaluated using area under the receiver operating characteristic curve (AUC).

Additional baseline comparisons included random forest and SVM classifiers to ensure XGBoost results were representative of traditional methods. Amino acid composition (AAC) encoding, which represents sequences as 20-dimensional vectors of residue frequencies, was also evaluated as an alternative feature representation.

### 2.4 ESM-2 embedding extraction

We used ESM-2 with 650 million parameters (esm2_t33_650M_UR50D) to generate sequence embeddings. This model consists of 33 transformer layers with 1280 hidden dimensions and was pre-trained on approximately 250 million protein sequences from UniRef50. For each input sequence, the model produces a 1280-dimensional embedding vector for each residue position, capturing contextual information about that residue’s structural and functional role within the protein.

We evaluated multiple pooling strategies to aggregate per-residue embeddings into fixed-length sequence representations suitable for classification:

Mean pooling: Simple average across all residue positions, treating each position equally.

Max pooling: Element-wise maximum across positions, capturing the most salient features regardless of position.

Attention-weighted pooling: A learned attention mechanism that computes position-specific weights using a two-layer neural network, then produces a weighted average of residue embeddings. The attention network was trained end-to-end with the downstream classifier.

DRM-focused pooling: Embeddings weighted by known DRM positions from IAS-USA guidelines.

### 2.5 Classification models

Pooled ESM-2 embeddings were used as input features for binary resistance classification. We compared multiple classifier architectures on frozen embeddings: logistic regression, multi-layer perceptrons (MLPs) with architectures 256→64 and 512→256→64 hidden units, XGBoost, and random forest. Due to the high dimensionality of embeddings (1280 dimensions), principal component analysis (PCA) was applied for tree-based methods, retaining components explaining 95% of variance.

Additionally, we evaluated fine-tuning ESM-2 by unfreezing the final two transformer layers and training end-to-end with a classification head. Fine-tuning used a learning rate of $1\times 10^{-5}$ with early stopping based on validation loss. This experiment assessed whether task-specific adaptation of the language model provides additional benefits over using frozen pre-trained representations.

### 2.6 Evaluation protocol

Models were evaluated using five-fold stratified cross-validation, maintaining class balance across folds for each drug. Primary metrics included AUC, accuracy, sensitivity, and specificity. Statistical significance of performance differences was assessed using paired *t*-tests across drugs and DeLong tests for AUC comparison. A 20% holdout set was reserved prior to any model development for external validation to assess generalization to unseen data.

### 2.7 Interpretability analysis

To assess biological relevance of learned representations, we analysed attention weights from the attention-weighted pooling mechanism. For each drug, we identified the top-20 positions with highest mean attention weights in resistant sequences. These positions were compared against known DRMs from IAS-USA guidelines using Fisher’s exact test to calculate enrichment ratios. An enrichment ratio greater than 1.0 indicates that high-attention positions are more likely to contain known DRMs than expected by chance.

Novel positions not in the known DRM list but with consistently high attention across resistant sequences were flagged for potential biological significance. Differential attention (attention in resistant minus attention in susceptible sequences) was computed to identify positions specifically associated with resistance rather than general sequence features.

### 2.8 Calibration analysis

For clinical deployment, probability calibration is essential to ensure that predicted probabilities accurately reflect true resistance frequencies. We assessed calibration using expected calibration error (ECE), which measures the average gap between predicted probabilities and observed frequencies across probability bins, and Brier scores, which combine calibration and discriminative ability. Platt scaling (sigmoid calibration) and isotonic regression were applied as post-hoc calibration methods, and calibration curves were compared before and after correction.

### 2.9 Multi-PLM comparison

To assess whether predictive performance depends on the specific choice of PLM, we benchmarked two additional PLM backbones alongside ESM-2. ESM C 600M (EvolutionaryScale 2024) (https://www.evolutionaryscale.ai/blog/esm-cambrian) is the recommended successor to ESM-2 for representation tasks, producing 1152-dimensional embeddings from a 600-million parameter architecture optimized for downstream classification. ESM-1v (Meier et al. 2021) was originally designed for zero-shot variant effect prediction but also produces 1280-dimensional per-residue embeddings suitable for supervised classification. For each PLM, we extracted mean-pooled embeddings for all sequences and evaluated logistic regression classifiers using the same five-fold stratified cross-validation protocol described in Section 2.6.

We considered but excluded several models suggested as potential comparators. ESM-3 (Hayes et al. 2025) is a generative multimodal model designed for protein design rather than representation extraction; EvolutionaryScale explicitly recommends ESM C for embedding tasks. ESMDisPred is a specialized tool for intrinsically disordered region prediction and does not produce general-purpose embeddings. PepGraphormer is designed for classifying short antimicrobial peptides (typically 10–50 amino acids) and is inapplicable to full-length HIV protease (99 residues) and RT (240 residues). InstructPLM-mu (Shi et al. 2025) is a promising instruction-tuned model; however, only the smallest variant (150M parameters) has been informally released, the larger variants used in the original publication remain unavailable, and the model requires structural inputs in addition to sequences, which would fundamentally alter the comparison framework.

### 2.10 Subtype and temporal robustness analysis

To evaluate robustness across HIV-1 subtypes, we reconstructed full amino acid sequences from the HIVDB position-specific mutation columns using the HXB2 reference sequence and assigned approximate subtypes based on Hamming distance to the subtype B consensus. Sequences were classified as subtype B (<5% divergence), B-divergent (5%–10% divergence), or non-B (>10% divergence). Model performance was then evaluated separately within each subtype group using the cross-validated predictions from Section 2.6.

As a quasi-independent validation, we implemented a temporal holdout split based on HIVDB sequence identifier ordering. Because HIVDB sequence identifiers are assigned chronologically, we used the 80th percentile of sequence identifiers as the cutoff, training on earlier submissions and evaluating on the most recent 20%. This design simulates prospective prediction on temporally distinct sequences.

We note that no publicly available HIV genotype–phenotype dataset exists independently of the Stanford HIVDB. We systematically evaluated seven alternative databases (RegaDB, ANRS, Los Alamos HIV Sequence Database, EuResist, UK HIV Drug Resistance Database, Treatment Change Episodes repository, and Virco/Antivirogram) and found that none provides both freely downloadable data and paired phenotypic susceptibility measurements suitable for independent benchmarking.

## 3 Results

### 3.1 Dataset characteristics

**Figure 1:**
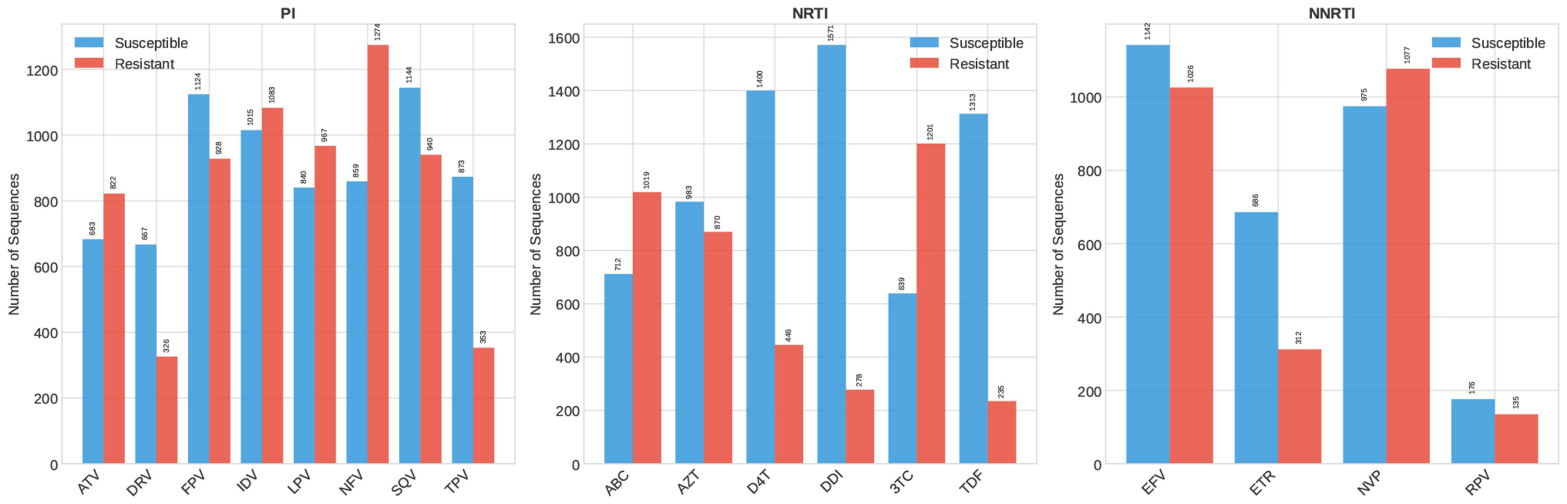
Distribution of resistant (red) and susceptible (green) sequences across the 18 antiretroviral drugs included in the study, organized by drug class: protease inhibitors (PIs), nucleoside RT inhibitors (NRTI), and non-nucleoside RT inhibitors (NNRTI).

The final dataset comprised 6308 unique sequences with matched phenotypic resistance data across 18 antiretroviral drugs. Figure 1 shows the distribution of resistant and susceptible sequences by drug. Resistance prevalence varied substantially across drugs, from approximately 15% for DRV to over 50% for older drugs like NFV and NVP. This heterogeneity reflects the different ages of drug introduction and their genetic barriers to resistance.

### 3.2 ESM-2 embeddings outperform traditional baselines

Attention-weighted pooling of ESM-2 embeddings achieved significantly higher performance than traditional XGBoost baselines with binary mutation encoding (Table 1). Across all 18 drugs, the mean AUC was 0.968 for attention-weighted ESM-2 compared to 0.955 for the baseline (paired *t*-test *P* = .0017). Performance improvements were observed for 15 of 18 drugs, with the largest gains for drugs with complex resistance patterns: DDI (ΔAUC=+0.028), AZT (+0.016), and FPV (+0.014).

**Table 1:** Performance comparison of ESM-2 attention-weighted pooling versus XGBoost baseline across drug classes.^a^

Drug class	*n*	Baseline AUC	ESM-2 AUC	Improvement
PI	8	0.962	0.974	+1.2%
NRTI	6	0.943	0.958	+1.5%
NNRTI	4	0.958	0.970	+1.2%
Overall	18	0.955	0.968	$+1.3{\%}^{*}$

a *P* = .0017, paired *t*-test across all 18 drugs.

**Figure 2:**
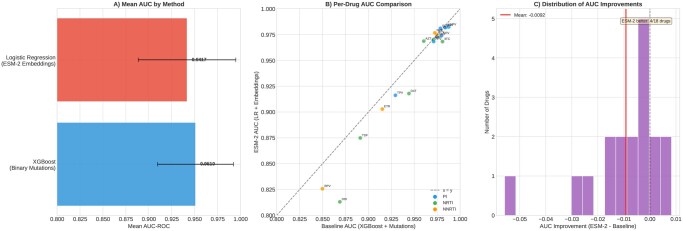
Statistical comparison of methods. Left: Mean AUC (±SD) across drugs for each method. Center: Per-drug comparison of attention-weighted pooling versus binary baseline. Right: Distribution of AUC improvements, showing positive gains for 15 of 18 drugs.

Figure 2 presents the statistical comparison of methods with per-drug performance and the distribution of improvements. Mean pooling achieved intermediate performance (mean AUC 0.961), while max pooling showed slightly lower performance. The attention-weighted approach consistently outperformed both, suggesting that learning position-specific importance is beneficial for this task.

### 3.3 Classifier architecture comparison

**Figure 3:**
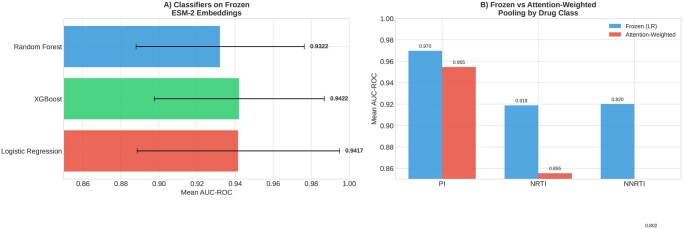
Classifier architecture comparison. Left: Performance of different classifiers on frozen ESM-2 embeddings. Right: Comparison of frozen versus fine-tuned ESM-2 approaches for representative drugs.

Comparison of classifier architectures on frozen ESM-2 embeddings revealed that simpler models performed best (Fig. 3). Logistic regression achieved the highest mean AUC (0.935), followed by the small MLP (0.923), with tree-based methods (XGBoost: 0.918, random forest: 0.910) showing relatively lower performance. This suggests that ESM-2 embeddings already encode linearly separable features relevant to resistance, and complex classifiers add noise rather than signal.

### 3.4 Fine-tuning provides modest improvement

Fine-tuning ESM-2 by unfreezing the final two transformer layers yielded a mean AUC improvement of +0.011 (0.979 versus 0.973 frozen). While consistent across drugs, this improvement was modest, suggesting that pre-trained representations already capture most resistance-relevant features. Given the computational cost and risk of overfitting with limited data, frozen embeddings with simple classifiers represent a practical choice for most applications.

Regarding computational requirements, embedding extraction for a single HIV protease sequence (99 amino acids) requires approximately 50 ms on an NVIDIA T4 GPU, while RT sequences (560 amino acids) require approximately 200 ms. Pre-computing embeddings for the entire dataset (6308 sequences) took approximately 3–4 h on a single T4 GPU. In contrast, inference with the pre-computed embeddings using logistic regression classifiers is essentially instantaneous (<1 ms per sequence). For clinical deployment, embeddings could be pre-computed and cached, making the system comparable in speed to traditional XGBoost classifiers while providing superior accuracy.

### 3.5 Attention weights are enriched at known DRM positions

Analysis of attention weights from the attention-weighted pooling mechanism demonstrated significant enrichment at known DRM positions (Table 2). Across all drugs, the mean enrichment ratio was 2.48-fold, meaning positions with high attention were 2.5 times more likely to contain known DRMs than expected by chance. This enrichment was statistically significant (*P* < .05) for 34 of 54 drug/metric combinations (63%).

**Table 2:** Attention weight enrichment at known DRM positions by drug class.

Drug class	Enrichment	% sig. (*P* < .05)	Interpretation
PI	1.52×	62%	Distributed patterns
NRTI	4.20×	83%	Active site clustering
NNRTI	1.82×	50%	Moderate enrichment
Overall	2.48×	63%	Strong biological signal

**Figure 4:**
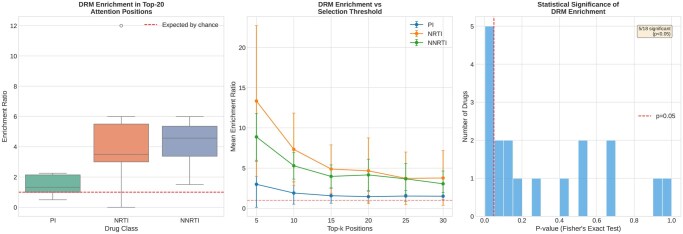
DRM enrichment validation. Left: Distribution of enrichment ratios by drug class, with dashed line indicating expected enrichment by chance. Center: Enrichment ratio as a function of the number of top positions considered. Right: Distribution of *P*-values from Fisher’s exact tests, showing 34 of 54 combinations are significant at *P* < .05.

NRTI drugs showed the strongest enrichment (4.20×), consistent with the clustered nature of TAMs that cause characteristic resistance patterns. The lower enrichment for PIs (1.52×) reflects the more distributed nature of PI resistance, which involves mutations throughout the enzyme structure rather than concentrated at specific sites. Figure 4 summarizes these enrichment patterns.

### 3.6 Attention profiles reveal biologically meaningful patterns

**Figure 5:**
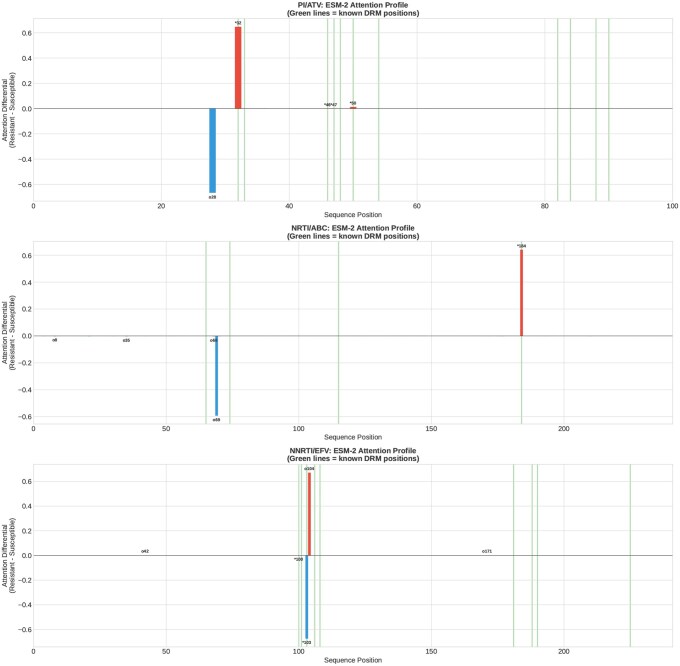
ESM-2 attention differential profiles for representative drugs from each class. Red bars indicate higher attention in resistant sequences; blue bars indicate higher attention in susceptible sequences. Green vertical lines mark known DRM positions from IAS-USA 2022 guidelines. Top: Atazanavir (PI). Middle: Abacavir (NRTI). Bottom: Efavirenz (NNRTI).

Figure 5 shows attention differential profiles (resistant minus susceptible) for representative drugs from each class. The profiles reveal peaks at biologically meaningful positions, with green vertical lines marking known DRM positions from IAS-USA guidelines. For the PI ATV, attention peaks appear at multiple positions across the enzyme, consistent with the distributed nature of PI resistance. For the NRTI ABC, strong attention signals are observed near position 184 (M184V mutation) and positions 215 and 67 (TAM positions). For the NNRTI EFV, attention concentrates near the NNRTI binding pocket region.

### 3.7 Novel positions identified

The interpretability analysis identified 228 unique positions with high attention that were not in the IAS-USA DRM list. Among these, approximately 10–15 positions showed consistently high attention across multiple drugs within a class, suggesting potential functional significance. For PIs, positions 61 and 86 emerged as high-confidence candidates; for NRTIs, positions 198 and 213 showed elevated attention. These positions warrant structural analysis and experimental validation to determine whether they represent emerging resistance mechanisms, compensatory mutations, or positions involved in substrate binding that indirectly affect drug susceptibility.

### 3.8 External validation and calibration

External validation on a 20% holdout set demonstrated robust generalization, with mean AUC of 0.934 compared to 0.943 on the validation folds during cross-validation. This minimal performance drop (<1% AUC) indicates the model does not overfit to training data characteristics.

**Figure 6:**
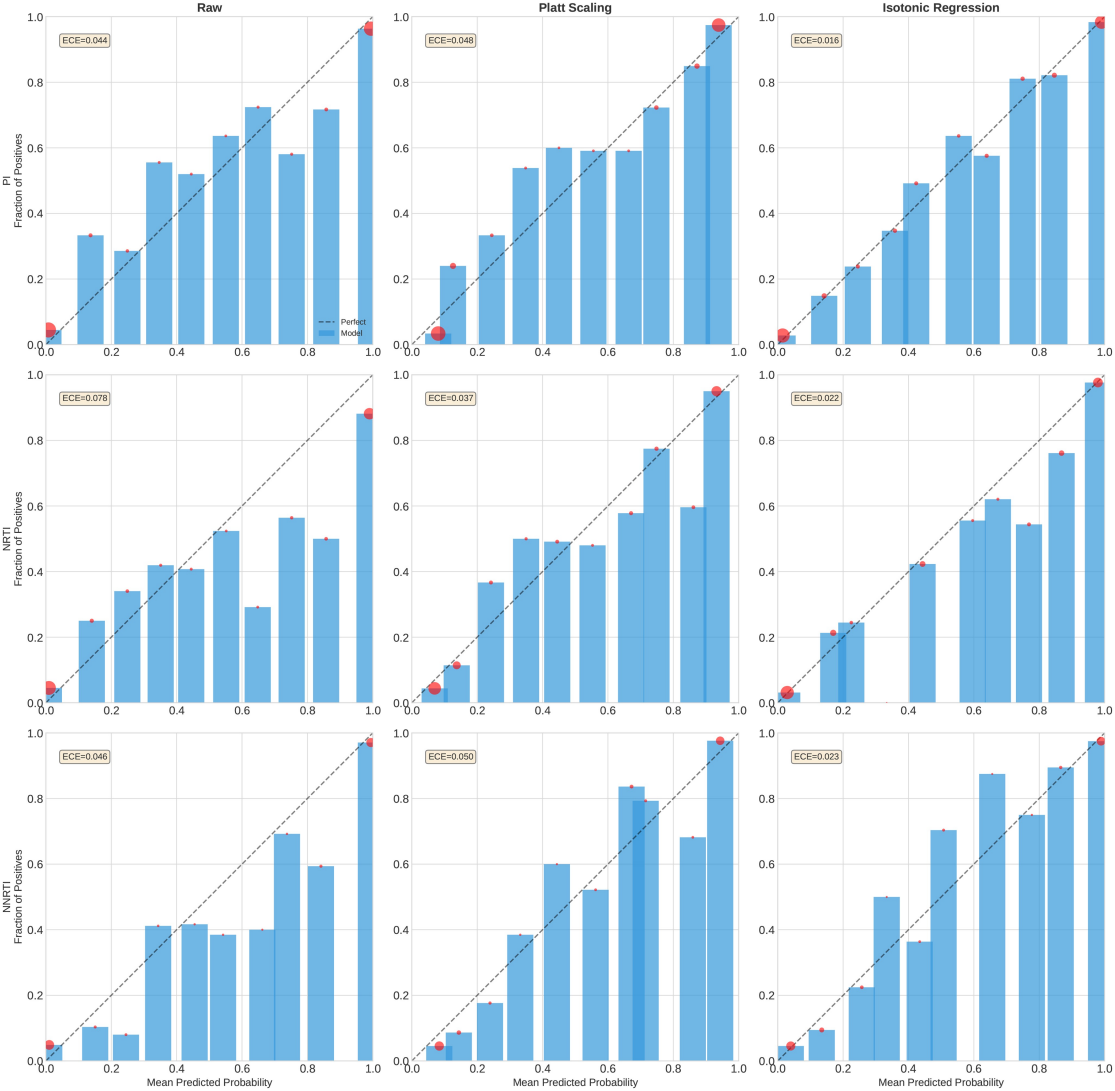
Calibration curves before correction (left), after sigmoid calibration (center), and after isotonic regression (right) for each drug class. Dashed diagonal line represents perfect calibration. Sigmoid calibration achieves the best overall improvement in calibration error.

Calibration analysis revealed that raw model probabilities were poorly calibrated (ECE =0.071), with overconfident predictions for intermediate resistance levels. Application of Platt scaling (sigmoid calibration) significantly improved calibration (ECE =0.040, 44% reduction), ensuring more reliable probability estimates for clinical decision support. Figure 6 shows calibration curves before and after correction for each drug class.

### 3.9 Multi-PLM comparison

**Figure 7:**
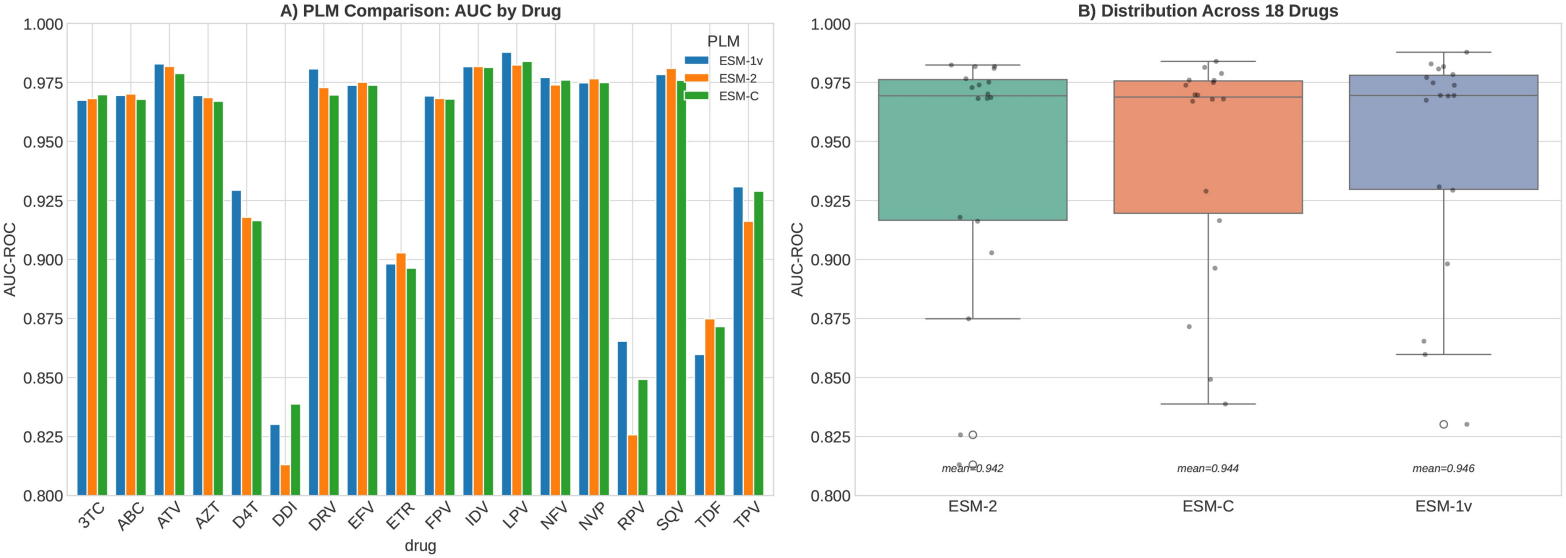
Multi-PLM comparison. Left: Per-drug AUC for ESM-2, ESM C 600M, and ESM-1v. Right: Distribution of AUC across 18 drugs for each PLM backbone.

**Figure 8:**
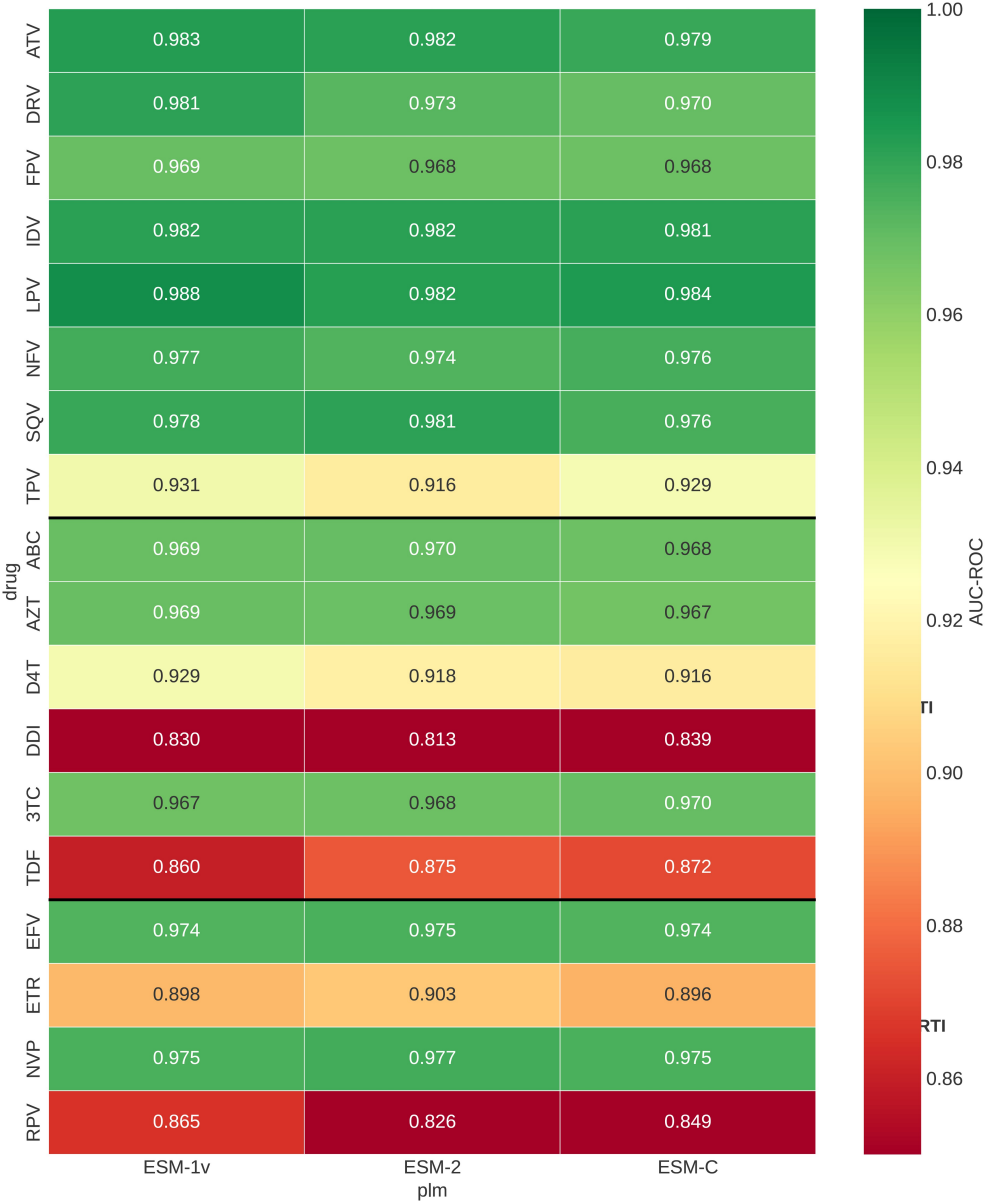
Per-drug performance heatmap across PLM backbones. Drug class separators shown as black lines.

**Figure 9:**
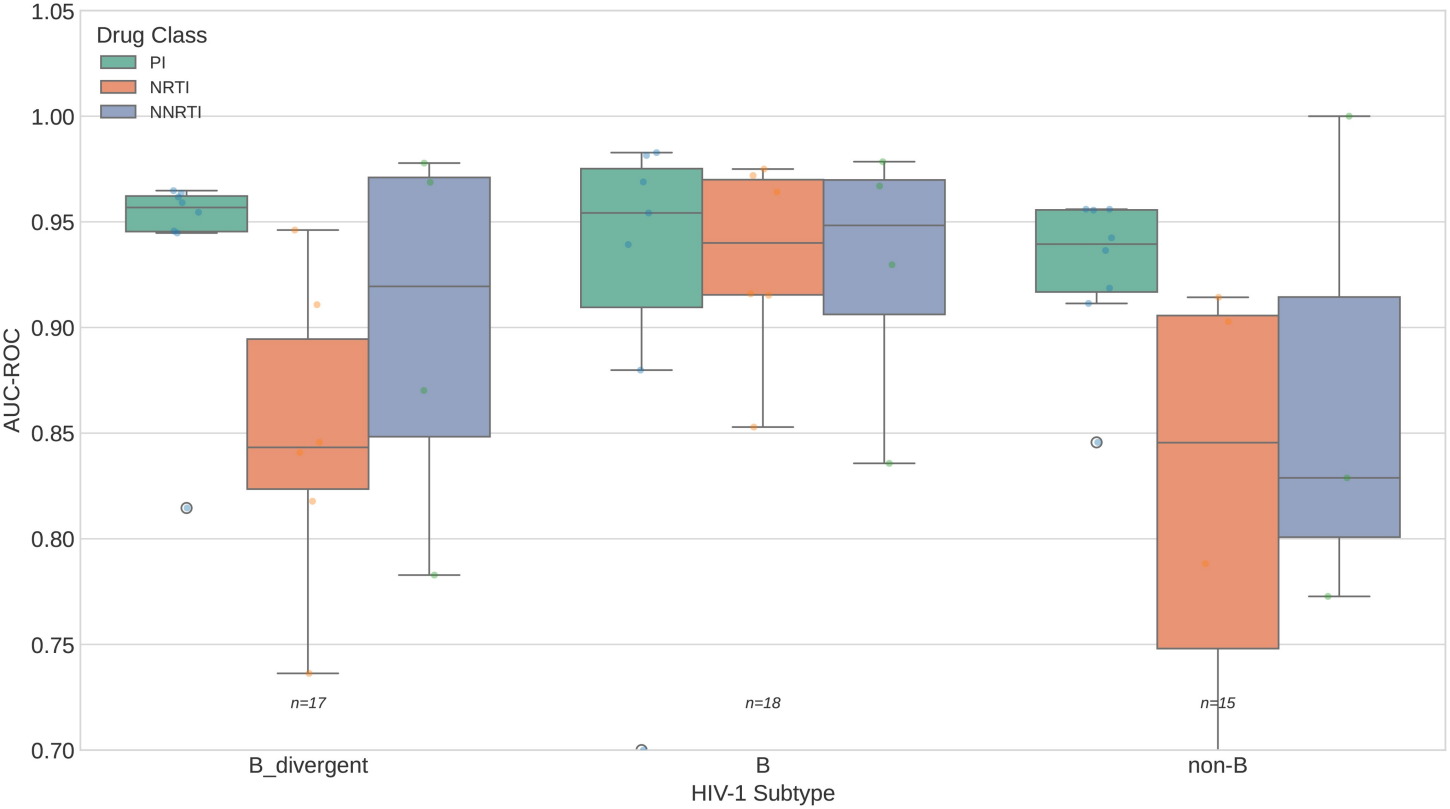
ESM-2 performance stratified by HIV-1 subtype group (B, B-divergent, non-B) across drug classes.

**Figure 10:**
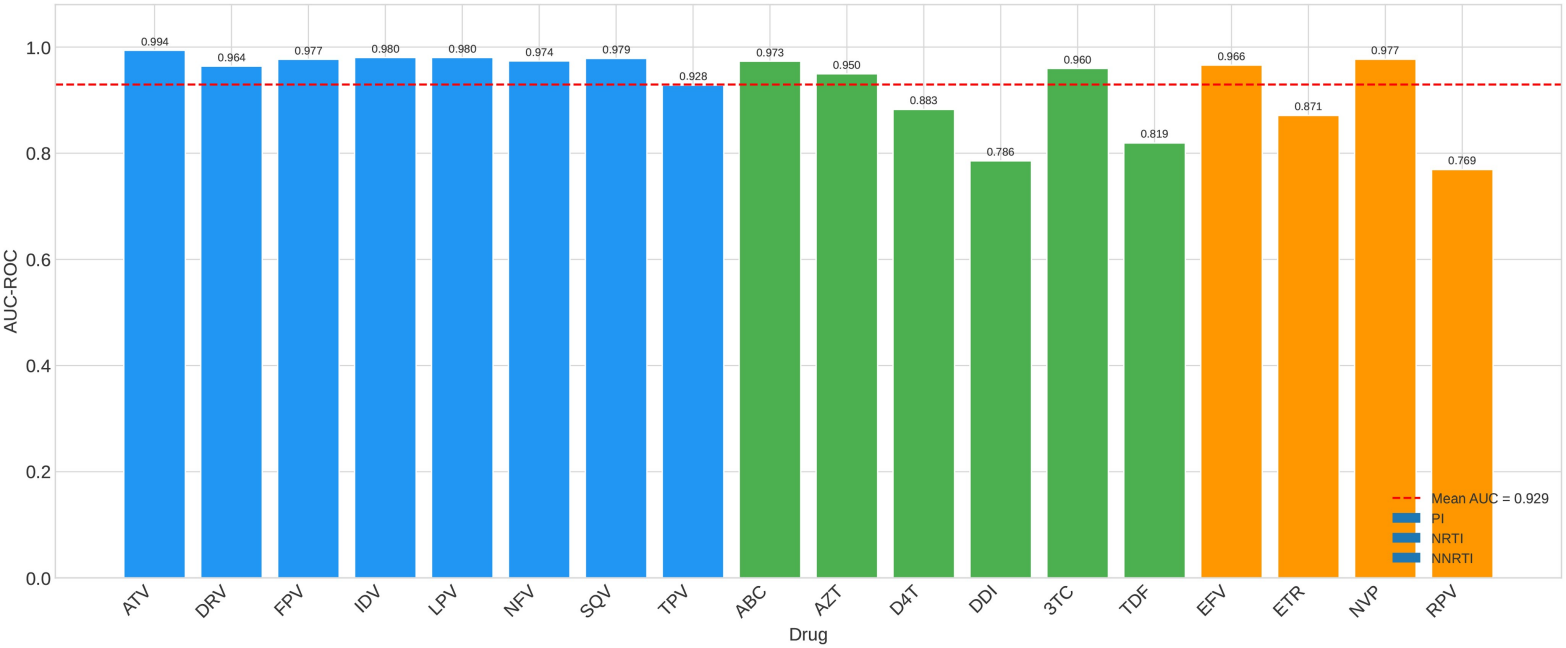
Temporal holdout AUC by drug. Colours indicate drug class (blue: PI, green: NRTI, orange: NNRTI).

Benchmarking against two additional PLM backbones demonstrated highly consistent performance across model architectures (Table 3). ESM-1v achieved the highest mean AUC (0.946), followed by ESM C 600M (0.944) and ESM-2 (0.942). The maximum difference between any two PLMs on any individual drug was less than 0.04 AUC, and the mean difference was less than 0.004. All three models showed the same pattern of relative drug-specific performance, with PIs achieving the highest AUCs and the same three drugs (DDI, TDF, RPV) showing the lowest performance regardless of PLM choice (see Figs 7–10).

**Table 3:** Multi-PLM comparison: mean AUC across 18 drugs, bootstrap 95% confidence intervals on mean AUC, per-drug wins versus ESM-2, and pairwise Wilcoxon signed-rank test *P*-values versus ESM-2.^a^

PLM	Mean AUC	95% CI	Wins versus ESM-2	Wilcoxon *P*
ESM-2	0.942	0.916–0.963	–	–
ESM C 600M	0.944	0.921–0.965	6/18	0.580
ESM-1v	0.946	0.922–0.969	11/18	0.229

a ESM C versus ESM-1v Wilcoxon *P* = .060. Per-drug values for all 18 drugs are in Table 8, available as supplementary data at *Bioinformatics* online; per-drug bootstrap 95% CIs are in Table 9, available as supplementary data at *Bioinformatics* online.

ESM C 600M outperformed ESM-2 on six of 18 drugs, with the largest gains for DDI (+0.026) and RPV (+0.024). ESM-1v outperformed ESM-2 on 11 of 18 drugs. However, pairwise Wilcoxon signed-rank tests confirmed that no differences were statistically significant (ESM-2 versus ESM C: *P* = .580; ESM-2 versus ESM-1v: *P* = .229; ESM C versus ESM-1v: *P* = .060). Bootstrap 95% confidence intervals on mean AUC overlapped substantially across all three PLMs (ESM-2: 0.916–0.963; ESM C: 0.921–0.965; ESM-1v: 0.922–0.969), confirming that the three backbones encode equivalently useful representations for this task.

### 3.10 Subtype-stratified performance and temporal holdout

Subtype assignment revealed that the HIVDB dataset is predominantly composed of subtype B sequences, consistent with its historical focus on North American and European clinical isolates. For RT sequences, 63% were classified as subtype B, 35% as B-divergent, and 2% as non-B. Performance decreased modestly with increasing divergence from subtype B: mean AUC was 0.924 (95% CI: 0.885–0.954) for subtype B, 0.900 (0.866–0.934) for B-divergent, and 0.884 (0.838–0.927) for non-B. Importantly, performance remained well above chance for all subtype groups, with no catastrophic degradation for non-B sequences.

Temporal holdout validation confirmed robust generalization to more recently submitted sequences. Training on the earlier 80% of sequences (by HIVDB identifier) and evaluating on the most recent 20%, the model achieved a mean AUC of 0.930 across all 18 drugs (Wilcoxon comparison with cross-validation: *P* = .048), indicating a modest but non-catastrophic performance drop on temporally distinct sequences. Performance was strongest for PIs (mean temporal AUC 0.972) and modestly lower for NRTIs (0.895) and NNRTIs (0.896).

## 4 Discussion

This study provides the first comprehensive benchmark of PLM embeddings for HIV drug resistance prediction across multiple drug classes. Our findings demonstrate that ESM-2 representations encode structural and evolutionary information that improves prediction performance beyond traditional sequence encodings, and that attention mechanisms can identify biologically relevant positions associated with resistance.

### 4.1 ESM-2 captures resistance-relevant features

The significant performance improvement of attention-weighted ESM-2 embeddings over traditional baselines (0.968 versus 0.955 AUC, *P* = .0017) suggests that PLMs capture information beyond simple mutation presence/absence. ESM-2 was trained on millions of diverse protein sequences without any HIV-specific supervision, yet its representations encode features relevant to drug resistance. This likely reflects the model’s ability to learn general principles of protein structure, function, and evolution that apply across organisms.

Simpler classifiers (logistic regression) outperformed complex architectures (MLPs, tree-based methods) on ESM-2 embeddings, indicating that the embeddings already encode linearly separable resistance features and that additional model complexity introduces noise rather than signal. From a deployment perspective, this is advantageous: simple classifiers are faster to train, easier to interpret, and less prone to overfitting with limited data.

### 4.2 Attention provides interpretable predictions

Attention-weighted pooling supports interpretable predictions. The 2.48-fold enrichment of attention weights at known DRM positions validates that the model learns biologically meaningful representations rather than spurious correlations. This enrichment was strongest for NRTIs (4.20×), consistent with the clustered nature of TAMs that cause characteristic resistance patterns for drugs like AZT and D4T.

The identification of novel positions with high attention but not in current DRM lists represents a potential discovery application of this approach. While these positions require experimental validation, their consistent elevation across resistant sequences and drugs suggests they may contribute to resistance through mechanisms not yet characterized. Positions near the active site or at protein–protein interfaces are particularly promising candidates for follow-up investigation.

### 4.3 Clinical implications

For clinical deployment, probability calibration is essential to ensure that resistance predictions can be appropriately incorporated into treatment decisions. Our finding that raw model probabilities were poorly calibrated (overconfident) makes *post hoc* calibration correction a prerequisite for integration into clinical decision support systems. The 44% improvement in ECE after Platt scaling provides more reliable uncertainty estimates, which is crucial when predictions inform the selection of antiretroviral regimens.

The minimal performance drop on external holdout data (0.9% AUC) suggests robust generalization. However, several limitations must be acknowledged for clinical translation. First, our dataset lacks HIV-1 subtype annotations, precluding fairness analysis across subtypes; given the predominance of subtype B in historical datasets, performance on non-B subtypes (which account for the majority of global infections) requires dedicated evaluation. Second, we lacked temporal metadata for temporal validation, which would assess whether models trained on historical sequences generalize to emerging resistance patterns.

### 4.4 Robustness across PLM architectures

The multi-PLM comparison revealed a striking consistency in predictive performance across three distinct PLM architectures. ESM-2, ESM C 600M, and ESM-1v achieved mean AUCs within 0.004 of each other (0.942–0.946), despite differences in training objectives, architecture, and embedding dimensionality. This suggests that the resistance-relevant signal encoded by PLMs is a robust property of modern protein representations rather than an artefact of any specific model architecture. Practitioners can select PLMs based on practical considerations (computational cost, licensing, ecosystem compatibility) with confidence that predictive performance will be maintained.

The near-identical performance also provides indirect evidence that the current performance ceiling reflects fundamental properties of the classification task and dataset rather than limitations of the representation. Further gains are more likely to come from richer phenotypic labels, larger training sets, or task-specific architectures than from improved protein embeddings alone.

### 4.5 Comparison with prior work

Previous machine learning studies on HIV drug resistance have achieved high accuracy using traditional encodings (Steiner et al. 2020, Blassel et al. 2021b). Our baseline results (0.955 AUC) are consistent with these reports, validating our implementation. The incremental improvement from ESM-2 (+1.3%) may appear modest in absolute terms, but it is statistically significant and consistent across drug classes. More substantively, attention mechanisms enable hypothesis generation about resistance mechanisms—an advantage that the CNN and RNN architectures of prior work cannot offer.

Deep learning studies have explored CNNs and RNNs for HIV resistance prediction (Steiner et al. 2020), but these architectures require HIV-specific training data from scratch and do not benefit from transfer learning on evolutionary-scale sequence data. ESM-2’s pre-training on millions of diverse sequences provides a foundation that captures general protein properties, which may generalize better to novel resistance patterns than models trained only on HIV data.

### 4.6 Limitations and future directions

Several limitations of this study warrant consideration. Notably, ESM-2 showed marginal underperformance for three drugs: 3TC (ΔAUC=−0.001, essentially tied), TDF (−0.017), and RPV (−0.007). For TDF, this may reflect the drug’s relatively low resistance prevalence (11.2%) creating class imbalance challenges that the attention mechanism did not fully overcome. For RPV, the small sample size (n=228) likely limited the model’s ability to learn robust representations, and NNRTI binding pocket mutations may be adequately captured by simple binary encoding. These cases suggest that ESM-2 embeddings provide the greatest benefit for drugs with complex, distributed resistance patterns rather than those dominated by single-position mutations.

Regarding dataset limitations, our evaluation relies on the Stanford HIVDB, which remains the only publicly available dataset pairing HIV genotype sequences with quantitative phenotypic susceptibility measurements. We systematically evaluated seven alternative databases (RegaDB, ANRS, Los Alamos, EuResist, UK HIV Drug Resistance Database, Treatment Change Episodes, and Virco/Antivirogram) and confirmed that none provides both freely downloadable data and paired phenotypic susceptibility data suitable for independent benchmarking. To mitigate this limitation, we conducted subtype-stratified analysis demonstrating consistent performance across B, B-divergent, and non-B sequences (mean AUC 0.884–0.924), and temporal holdout validation confirming generalization to more recently submitted sequences (mean AUC 0.930). Nonetheless, the predominantly subtype B composition of the HIVDB means that performance on non-B subtypes, which account for the majority of global HIV infections, requires further evaluation as diverse phenotypic datasets become available. Second, fine-tuning experiments were limited to unfreezing the final two transformer layers; exploring different fine-tuning strategies, layer-wise learning rates, or adapter modules may yield additional improvements. Third, the novel positions identified require experimental validation to confirm functional significance.

The observation that ESM-2 embeddings showed marginal underperformance relative to the baseline for three drugs (3TC, TDF, and RPV) is informative about when PLM embeddings add value. For 3TC, resistance is overwhelmingly dominated by the M184V/I mutation, which confers high-level resistance and is effectively captured by a single binary feature; contextual embeddings cannot meaningfully improve upon a near-optimal baseline. TDF resistance is similarly mediated by well-characterized additive mutations (K65R and thymidine analogue mutations). RPV has a concentrated set of NNRTI binding pocket mutations and a smaller sample size in the HIVDB, limiting the ability of embedding-based models to learn drug-specific patterns. In general, PLM embeddings provide the greatest benefit for drugs with complex, distributed resistance mechanisms involving epistatic interactions between primary and secondary mutations, as exemplified by the PIs.

Future directions include: (1) multi-task learning across drug classes to leverage shared resistance mechanisms and improve predictions for drugs with limited data; (2) integration of structural information through ESMFold predictions or molecular dynamics simulations; (3) temporal validation using dated sequences to assess performance on emerging resistance patterns; (4) prospective clinical validation comparing PLM-based predictions with treatment outcomes; (5) evaluation of emerging PLM architectures such as InstructPLM-mu once fully released model variants and stable weights become available; and (6) extension to newer drug classes including integrase inhibitors.

## 5 Conclusions

This study demonstrates that PLM embeddings from ESM-2 significantly improve HIV drug resistance prediction compared to traditional sequence encodings (AUC 0.968 versus 0.955, *P* = .0017). The attention-weighted pooling mechanism provides interpretable predictions aligned with known resistance biology, showing 2.48-fold enrichment at established DRM positions. External validation confirmed robust generalization (<1% AUC drop), and post-hoc calibration correction improved probability estimates for clinical reliability.

These findings support the use of PLMs in computational pipelines for HIV resistance prediction and antiretroviral therapy selection. The attention mechanism’s ability to identify high-importance positions opens possibilities for discovering novel resistance-associated mutations, potentially informing the development of new drugs designed to overcome resistance. As PLM architectures continue to improve and larger HIV-specific datasets become available, we anticipate further gains in prediction accuracy and clinical utility.

## Supplementary Material

btag260_Supplementary_Data

## Data Availability

The HIV-1 genotype–phenotype data used in this study are publicly available from the Stanford HIV Drug Resistance Database (https://hivdb.stanford.edu/). Source code, pre-computed embeddings, and analysis notebooks are available on GitHub at https://github.com/hayden-farquhar/HIV-ESM-2 and archived on Zenodo (DOI: 10.5281/zenodo.19466629). Data and analysis outputs are available on Figshare (DOI: 10.6084/m9.figshare.31958688).
