# Supplementary material for: Protein language model embeddings improve HIV drug resistance prediction: a comprehensive benchmark with attention-based interpretability
Source: Bioinformatics. 2026 May 9;42(5):btag260. doi: 10.1093/bioinformatics/btag260 (PMC13198382; doi:10.1093/bioinformatics/btag260)
Supplement: btag260_Supplementary_Data [file btag260_supplementary_data.docx]

**Supplementary Materials**

Protein Language Model Embeddings Improve HIV Drug Resistance Prediction: A Comprehensive Benchmark with Attention-Based Interpretability

Hayden Farquhar, MBBS MPHTM

*Independent Researcher | hayden.farquhar@icloud.com | ORCID: 0009-0002-6226-440X*

# **Contents**

Table S1: Per-drug performance metrics for all 18 antiretroviral drugs

Table S2: Dataset composition and class balance

Table S3: Classifier architecture comparison results

Table S4: DRM enrichment statistics by drug class

Table S5: External validation results

Table S6: Calibration metrics before and after correction

Table S8: Multi-PLM comparison (ESM-2, ESM C 600M, ESM-1v)

Table S9: Per-drug bootstrap 95% confidence intervals

Table S10: Subtype-stratified performance

Table S11: Temporal holdout validation results

Table S7: Novel positions with high attention weights

# **Supplementary Table S1**

*Per-drug performance metrics for all 18 antiretroviral drugs. AUC values from 5-fold cross-validation comparing attention-weighted ESM-2 pooling versus XGBoost with binary mutation encoding. Bold Δ AUC values indicate ESM-2 improvement over baseline.*

| **Class** | **Drug** | **N samples** | **% Resistant** | **Baseline AUC** | **ESM-2 AUC** | **Δ AUC** |
| --- | --- | --- | --- | --- | --- | --- |
| PI | ATV | 1,505 | 52.8% | 0.983 | 0.986 | **+0.004** |
| PI | DRV | 993 | 31.0% | 0.973 | 0.983 | **+0.007** |
| PI | FPV | 2,052 | 43.0% | 0.972 | 0.978 | **+0.014** |
| PI | IDV | 2,098 | 54.6% | 0.986 | 0.988 | **+0.004** |
| PI | LPV | 1,807 | 49.0% | 0.987 | 0.991 | **+0.004** |
| PI | NFV | 2,133 | 57.0% | 0.987 | 0.989 | **+0.003** |
| PI | SQV | 2,084 | 49.9% | 0.983 | 0.989 | **+0.005** |
| PI | TPV | 1,226 | 49.5% | 0.926 | 0.954 | **+0.007** |
| NRTI | 3TC | 1,840 | 48.7% | 0.984 | 0.985 | -0.001 |
| NRTI | ABC | 1,731 | 54.5% | 0.962 | 0.967 | **+0.002** |
| NRTI | AZT | 1,853 | 54.0% | 0.963 | 0.977 | **+0.016** |
| NRTI | D4T | 1,846 | 53.3% | 0.956 | 0.958 | **+0.012** |
| NRTI | DDI | 1,849 | 33.0% | 0.887 | 0.909 | **+0.028** |
| NRTI | TDF | 1,548 | 11.2% | 0.913 | 0.931 | -0.017 |
| NNRTI | EFV | 2,270 | 51.5% | 0.977 | 0.988 | **+0.010** |
| NNRTI | ETR | 1,298 | 77.0% | 0.928 | 0.947 | **+0.012** |
| NNRTI | NVP | 337 | 67.4% | 0.974 | 0.987 | **+0.005** |
| NNRTI | RPV | 228 | 52.6% | 0.853 | 0.916 | -0.007 |

*Note: ESM-2 outperformed baseline on 15 of 18 drugs. Three drugs showed minimal underperformance: 3TC (-0.001, essentially tied), TDF (-0.017), and RPV (-0.007).*

# **Supplementary Table S2**

*Dataset composition by drug class showing target protein, unique sequences, and number of drugs tested.*

| **Drug Class** | **Target Protein** | **Unique Sequences** | **Number of Drugs** |
| --- | --- | --- | --- |
| Protease Inhibitors (PI) | HIV-1 Protease | 2,171 | 8 |
| NRTIs | Reverse Transcriptase | 1,867 | 6 |
| NNRTIs | Reverse Transcriptase | 2,270 | 4 |
| **Total** | — | **6,308** | **18** |

# **Supplementary Table S3**

*Classifier architecture comparison on frozen ESM-2 embeddings. Mean AUC across all 18 drugs. PCA was applied to embeddings for tree-based methods to handle high dimensionality.*

| **Classifier** | **Mean AUC** | **Std Dev** | **Rank** |
| --- | --- | --- | --- |
| **Logistic Regression** | **0.935** | 0.031 | 1 |
| MLP (256→64) | 0.923 | 0.038 | 2 |
| XGBoost (PCA) | 0.918 | 0.042 | 3 |
| Random Forest (PCA) | 0.910 | 0.045 | 4 |

# **Supplementary Table S4**

*DRM enrichment analysis by drug class. Enrichment ratio calculated as the ratio of observed DRMs in top-20 attention positions to expected by chance based on known IAS-USA 2022 DRM positions.*

| **Drug Class** | **Known DRMs** | **Enrichment Ratio** | **Significant (p<0.05)** | **Interpretation** |
| --- | --- | --- | --- | --- |
| PI | 46 | 1.52x | 62% | Distributed patterns |
| NRTI | 34 | **4.20x** | 83% | Active site clustering |
| NNRTI | 19 | 1.82x | 50% | Moderate enrichment |
| **Overall** | **99** | **2.48x** | **63%** | **Strong biological signal** |

# **Supplementary Table S5**

*External validation results comparing internal cross-validation performance to held-out test set (20% of data).*

| **Metric** | **Internal (CV)** | **Holdout (20%)** | **Drop** |
| --- | --- | --- | --- |
| Mean AUC | 0.943 | **0.934** | 0.9% |
| Mean Accuracy | 0.912 | 0.905 | 0.7% |

# **Supplementary Table S6**

*Calibration metrics before and after post-hoc correction. ECE = Expected Calibration Error. Lower values indicate better calibration.*

| **Metric** | **Before Correction** | **After (Sigmoid)** | **Improvement** |
| --- | --- | --- | --- |
| ECE (Expected Calibration Error) | 0.071 | **0.040** | **44%** |
| Brier Score | 0.086 | 0.075 | 13% |

# **Supplementary Table S7**

*Summary of novel positions with high attention weights not present in IAS-USA 2022 DRM list. These positions represent candidates for experimental validation.*

| **Drug Class** | **Novel Positions** | **High-Confidence Candidates** | **Notes** |
| --- | --- | --- | --- |
| PI | 50 | Positions 61, 86 | Near active site |
| NRTI | 93 | Positions 198, 213 | Elevated attention |
| NNRTI | 85 | Multiple candidates | Requires validation |
| **Total** | **228** | **~10-15 strong** | — |

**Supplementary Table S8**

Multi-PLM comparison: per-drug AUC (5-fold cross-validation, logistic regression). Pairwise Wilcoxon signed-rank tests: ESM-2 vs ESM C p=0.580; ESM-2 vs ESM-1v p=0.229; ESM C vs ESM-1v p=0.060.

| **Drug** | **Class** | **ESM-2** | **ESM C 600M** | **ESM-1v** |
| --- | --- | --- | --- | --- |
| ATV | PI | 0.982 | 0.979 | 0.983 |
| DRV | PI | 0.973 | 0.970 | 0.981 |
| FPV | PI | 0.968 | 0.968 | 0.969 |
| IDV | PI | 0.982 | 0.981 | 0.982 |
| LPV | PI | 0.982 | 0.984 | 0.988 |
| NFV | PI | 0.974 | 0.976 | 0.977 |
| SQV | PI | 0.981 | 0.976 | 0.978 |
| TPV | PI | 0.916 | 0.929 | 0.931 |
| ABC | NRTI | 0.970 | 0.968 | 0.970 |
| AZT | NRTI | 0.969 | 0.967 | 0.970 |
| D4T | NRTI | 0.918 | 0.917 | 0.929 |
| DDI | NRTI | 0.813 | 0.839 | 0.830 |
| 3TC | NRTI | 0.968 | 0.970 | 0.968 |
| TDF | NRTI | 0.875 | 0.872 | 0.860 |
| EFV | NNRTI | 0.975 | 0.974 | 0.974 |
| ETR | NNRTI | 0.903 | 0.896 | 0.898 |
| NVP | NNRTI | 0.977 | 0.975 | 0.975 |
| RPV | NNRTI | 0.826 | 0.849 | 0.865 |
| Mean |  | 0.942 | 0.944 | 0.946 |

**Supplementary Table S9**

Per-drug bootstrap 95% confidence intervals for ESM-2 logistic regression (1000 bootstrap iterations).

| **Drug** | **Class** | **AUC** | **95% CI Lower** | **95% CI Upper** |
| --- | --- | --- | --- | --- |
| ATV | PI | 0.982 | 0.976 | 0.987 |
| DRV | PI | 0.973 | 0.963 | 0.981 |
| FPV | PI | 0.968 | 0.962 | 0.975 |
| IDV | PI | 0.982 | 0.977 | 0.986 |
| LPV | PI | 0.982 | 0.977 | 0.988 |
| NFV | PI | 0.974 | 0.967 | 0.981 |
| SQV | PI | 0.981 | 0.976 | 0.986 |
| TPV | PI | 0.916 | 0.898 | 0.933 |
| ABC | NRTI | 0.970 | 0.964 | 0.976 |
| AZT | NRTI | 0.969 | 0.961 | 0.976 |
| D4T | NRTI | 0.918 | 0.904 | 0.932 |
| DDI | NRTI | 0.813 | 0.781 | 0.842 |
| 3TC | NRTI | 0.968 | 0.958 | 0.977 |
| TDF | NRTI | 0.875 | 0.847 | 0.902 |
| EFV | NNRTI | 0.975 | 0.969 | 0.981 |
| ETR | NNRTI | 0.903 | 0.880 | 0.923 |
| NVP | NNRTI | 0.977 | 0.970 | 0.983 |
| RPV | NNRTI | 0.826 | 0.772 | 0.868 |

**Supplementary Table S10**

Subtype-stratified ESM-2 performance. Subtypes assigned via Hamming distance to HXB2 reference. Bootstrap 95% CIs from 1000 iterations.

| **Subtype** | **Mean AUC** | **95% CI Lower** | **95% CI Upper** | **n pairs** |
| --- | --- | --- | --- | --- |
| B | 0.924 | 0.885 | 0.954 | 17 |
| B-divergent | 0.900 | 0.866 | 0.934 | 18 |
| Non-B | 0.884 | 0.838 | 0.927 | 15 |

**Supplementary Table S11**

Temporal holdout validation. Train: SeqID <= 80th percentile; Test: most recent 20%. Wilcoxon comparison with cross-validation: p=0.048.

| **Drug** | **Class** | **AUC** | **n train** | **n test** |
| --- | --- | --- | --- | --- |
| ATV | PI | 0.994 | 1071 | 434 |
| DRV | PI | 0.964 | 561 | 432 |
| FPV | PI | 0.977 | 1618 | 434 |
| IDV | PI | 0.980 | 1664 | 434 |
| LPV | PI | 0.980 | 1373 | 434 |
| NFV | PI | 0.974 | 1699 | 434 |
| SQV | PI | 0.979 | 1650 | 434 |
| TPV | PI | 0.928 | 793 | 433 |
| ABC | NRTI | 0.973 | 1369 | 362 |
| AZT | NRTI | 0.950 | 1479 | 374 |
| D4T | NRTI | 0.883 | 1484 | 362 |
| DDI | NRTI | 0.786 | 1487 | 362 |
| 3TC | NRTI | 0.960 | 1466 | 374 |
| TDF | NRTI | 0.819 | 1174 | 374 |
| EFV | NNRTI | 0.966 | 1742 | 426 |
| ETR | NNRTI | 0.871 | 579 | 419 |
| NVP | NNRTI | 0.977 | 1662 | 390 |
| RPV | NNRTI | 0.769 | 273 | 38 |
| Mean |  | 0.930 |  |  |
